# Role of Fungal Infections in Carcinogenesis and Cancer Development: A Literature Review

**DOI:** 10.34172/apb.2022.076

**Published:** 2021-09-29

**Authors:** Kamran Hosseini, Hossein Ahangari, Florence Chapeland-leclerc, Gwenael Ruprich-Robert, Vahideh Tarhriz, Azita Dilmaghani

**Affiliations:** ^1^Department of Molecular Medicine, Faculty of Advanced Medical Sciences and Technologies, Shiraz University of Medical Sciences, Shiraz, Iran.; ^2^Student Research Committee, Shiraz University of Medical Sciences, Shiraz, Iran.; ^3^Department of Food Science and Technology, Faculty of Nutrition and Food Science, Tabriz University of Medical Sciences, Tabriz, Iran.; ^4^Université de Paris, Faculté des Sciences, Laboratoire Interdiciplinaire des Energies de Demain (LIED), UMR 8236 CNRS, F-75013, Paris, France.; ^5^Molecular Medicine Research Center, Bio-Medicine Institute, Tabriz University of Medical Sciences, Tabriz, Iran.; ^6^Drug Applied Research Center, Tabriz University of Medical Sciences, Tabriz, Iran.; ^7^Department of Pharmaceutical Biotechnology, Faculty of Pharmacy, Tabriz University of Medical Sciences, Tabriz, Iran.

**Keywords:** Cancer, Fungi, Infection, Carcinogenesis, Fungal species

## Abstract

Cancer is a serious debilitating disease and one of the most common causes of death. In recent decades the high risk of various cancers enforced scientists to discover novel prevention and treatment methods to diminish the mortality of this terrifying disease. Accordingly, its prevention can be possible in near future. Based on epidemiological evidence, there is a clear link between pathogenic fungal infections and cancer development. This association is often seen in people with weakened immune systems such as the elderly and people with acquired immunodeficiency (AIDS). Carcinoma in these people is first seen chronically and then acutely. Although the different genetic and environmental risk factors are involved in carcinogenesis, one of the most important risk factors is fungal species and infections associating with cancers etiology. Now it is known that microbial infection is responsible for initiating 2.2 million new cancer cases. In this way, many recent studies have focused on investigating the role and mechanism of fungal infections in diverse cancers occurrence. This review provides a comprehensive framework of the latest clinical findings and the association of fungal infections with versatile cancers including esophageal, gastric, colorectal, lung, cervical, skin, and ovarian cancer.

## Introduction

Diseases triggered by excessively dividing of the tissue cells that can develop into intrusive lumpy masses is called cancer, which also is mentioned to as tumors. About 200 types of cancers in human kind are currently established by the National Cancer Institute (http://www.cancer.gov/types) and among them, some cancers can spread from their origin to other body tissues in a process so-called metastasis. Cancer classification is based on the tissue and/or organ of the origin e.g. carcinomas that refers to the cancers which arise in tissues or cover body organs. Also, other classes like melanomas, sarcomas, leukemia and lymphomas, are other known kinds of cancers.^1^

The cancer Tsunami is going to be more expanded. For instance, 150 000 new cases of colorectal cancers (CRC) are identified annually in the US. So, the studies have been focused on exploring the risk factors of different cancers to drop the incidence of them. Every kind of the cancers is affected by special risk factors, e.g., history of inflammatory bowel disease (IBD), type 2 diabetes and family history that are the main risk factors of CRC.^2,3^ It is noticeable that there will be increased incidence, approximately 50%, and near 17 million deaths by 2030 and this fact stress the necessity of discovering the more selective treatments for cancers based on their originating factor especially for the cancers triggered by microbial infections to diminish the unintended harmful outcomes on healthy tissues.^4^

**Figure 1:**
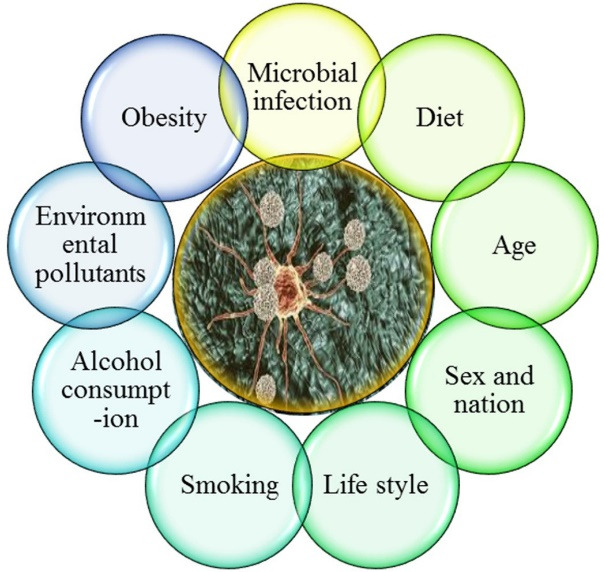
Different types of risk factors in carcinogenesis

Risk factors involved in cancers can vary by the area and country parallel to overall cancer incidence and mortality arises. Factors including age, smoking, diet and fat saturated regimes, body weight, alcohol consumption, exposure to pollutants and/or radioactivity, genetic background, and the infection by bacterial and fungal species have been coupled to the risk of carcinogenesis and versatile types of cancers (Figure 1). The studies revealed that infection by microbial species as a risk factor, is responsible for initiating of 2.2 million new cancer cases.^5^ Nowadays, it is estimated that around 16% of the overall cancer incidences is associated to microbial infections and toxicities. On the other hand, it is proposed that the microbial infection not only can boost the cancer risk but can also assist its treatment.^6^

**Figure 2:**
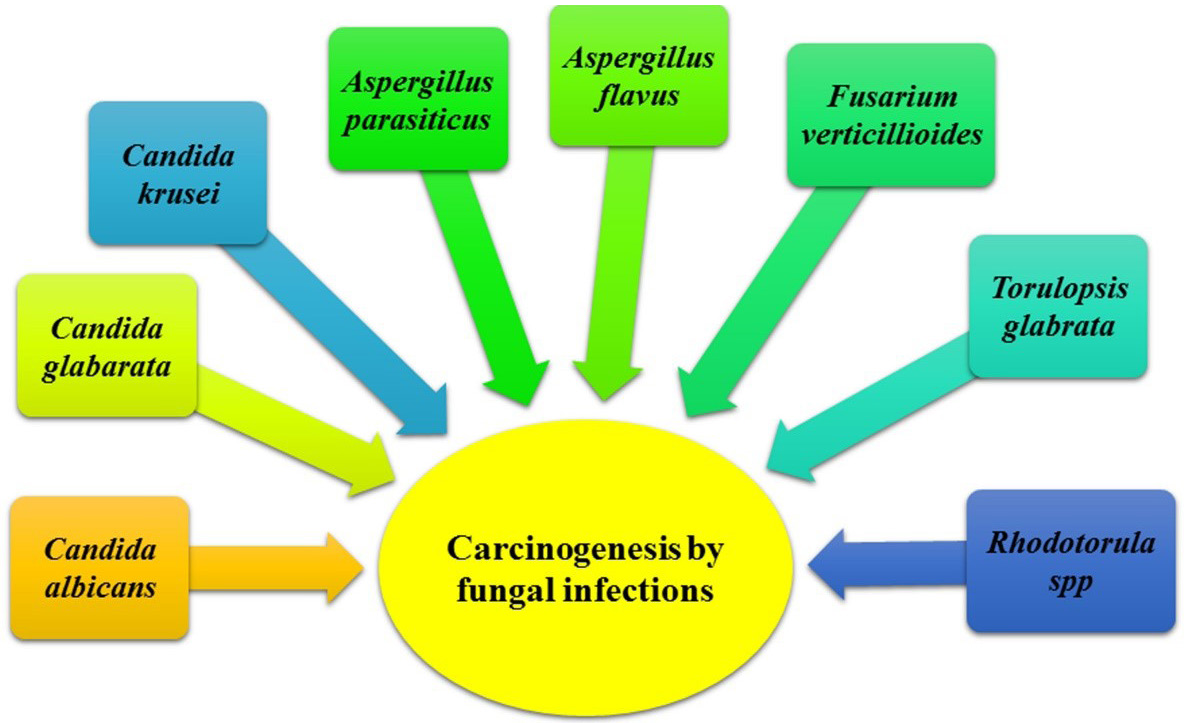
Different studied fungal genera and species in carcinogenesis.

Invasive fungal infections (IFIs) maintain to pose a challenge in cancer development and are linked with a high incidence of mortality and morbidity. By the last 20 years, despite the development of numerous diagnostics and therapeutic in the field of fungal infections, the IFI mortality has sustained to 35.7% growth. This means that the cancer growth rate is probably affected by IFI and it’s needed to be scrutinized.^7^ The most common genera and species of fungi involved in cancers are *Candida albicans*, *C. glabrata*, *C. tropicalis*, *Aspergillus flavus*, *A. parasiticus*, *Fusarium verticillioides* and *F. proliferatum.* Nevertheless, continuous efforts to distinguish the association between the cancers and fungal infections should be nonstop since it is not expected that the combat against cancer to be won soon (Figure 2).^8^

 In the present review, the role of opportunistic and pathogenic fungal infections with carcinogenesis of various organs, especially solid organs has been reviewed based on the latest clinical research studies and the most up-to-date scientific articles published in databases including PubMed and Scopus. Before this review, there was no complete report on the relationship between fungi and carcinogenesis of solid organs; however, the current study has thoroughly investigated the gap between the role of different fungi and carcinogenesis.

## The role of fungal infections in esophageal cancer

Esophageal carcinoma is the sixth leading cause of cancer-related mortality and the eighth leading cause of cancer worldwide. The disease is more prevalent in men,^9^ but in most countries of the world, the incidence rate per 100 000 people affects 2.5-5 men and 1.5-2.5 women.^10^ Histologically, the disease is classified into two categories: esophageal adenocarcinoma, which affects the lower esophagus, and esophageal squamous cell carcinoma, which affects the middle and upper esophagus.^11^ Numerous risk factors for this type of cancer have been reported, including smoking, alcohol consumption, consumption of hot beverages and foods, poor nutrition, exposure to chemicals, environmental pollutants and radiation, and exposure to pathogenic and opportunistic microorganisms.^12-14^ In addition, various fungal infections can arise for esophageal carcinoma, the most common fungus is *Candida* spp., which is an opportunistic yeast that colonizes the mucosa of the genitourinary and gastrointestinal tract of healthy people (about 30%-50% of people) without causing infection. *Candida* mostly occurs during the immune system suppression which manifests by skin, nails, and mucosa (esophagus) signs. In relation to mucus, this yeast causes chronic mucocutaneous candidiasis disease (CMCD).^15^ The most common genera and species of candida associated with candidiasis are the following: *Candida albicans*, *C. glabrata*, *C. tropicalis,*^16^ *C. krusei*, *C. parapsilosis*, *Torulopsis glabrata*, *T. tomato*,* Aspergillus flavus*, *A. parasiticus*, *Fusarium verticillioides*, *F. proliferation.*^17-20^ According to a study by Koo et al, in patients with primary immunodeficiency, esophageal candidiasis occurs in cancer patients as CMCD with persistent or recurrent thrush. CMCD is caused by at least five mutations that ultimately affect the IL-17 pathways; One of these is a mutation in the autosomal dominant gene gain of function of the STAT 1 signaling protein, in which a genetic defect intensifies Th1 immune responses and ultimately reduces the production of IL-17 and IL-22.^21^ In another study, Domingues-Ferreira et al examined a patient with chronic mucocutaneous candidiasis who had decades of treatment-resistance esophageal candidiasis and eventually developed epidermoid esophageal cancer. In this patient, modulation of immune function induced by mitogen suppression and antigen-induced lymphoproliferation disrupted NK cytotoxic activity and IL-2 secretion, resulting in increased IL-4 secretion and lymphocyte apoptosis.^22^

## The role of fungal infections in gastric cancer

Gastric cancer (GC) is the fourth leading cause of malignancy and the second leading cause of mortality in all malignancies worldwide.^23,24^ The five-year survival rate is 90% in Japan and 10%-30% in European countries. Due to the incidence of GC, more than 50% of new cases occur in developing countries.^25,26^ GC is generally classified into four categories: Sporadic gastric cancer (SGCs),^27^ Early onset gastric cancer (EOGC),^27^ gastric stump cancer (GSC),^28^ and hereditary diffuse gastric cancer (HDGC).^29^ Risk factors for this cancer include smoking,^30^ alcohol consumption,^31^ obesity,^32^ pernicious anemia and blood type A,^33^ mycotoxins^34^ and *Helicobacter pylori* infection.^35^

Preliminary studies based on culture-dependent methods have shown that fungi make up about 70% of the digestive system of adults and their number is in the range of 10^2^.^36^ The presence of some fungi can contribute to development of GC, including *C. albicans,*^37^ *Phialemonium* spp.,^38^ *Aspergillus* spp., *Penicillium* spp., and *Fusarium* spp.^39^ But in general, number of commensal fungal species are predominant in the gastrointestinal tract, including *S. cerevisiae*, *Malassezia* spp., and some species are classified as transient microbiota, e.g., *Aspergillus* spp., *Mucor* spp., *Cryptococcus* spp., *Rhodotorula* spp., *Trichosporon* spp., *Histoplasma* spp., *Coccidioides* spp., *Paracoccidioides* spp. and *Blastomyces* spp.^40,41^ Among these fungi, there is ample evidence that *C. albicans* is able to initiate and develop cancerous events through several mechanisms: (1) Production of carcinogens such as acetaldehyde, which contribute to the progression of cancer (*C. albicans, C. tropicalis, C. parapsilosis* produce more acetaldehyde than other species of Candida).^41^ (2) Induction of inflammatory processes that contributes to tumor metastasis (using cytokines such as CXCL1, CXCL2, CXCL3, TNF-α, and IL-18 facilitates tumorigenesis, angiogenesis, and metastasis).^34,42-45^ (3) Processes related to molecular mimicry (molecular mimic of the protein associated with complement receptor 3 (CR3-CP) *C. albicans* that supports cancer progression).^46^ (4) The Th17 response (Th17 is a subset of CD4 + T cells that are active in response against *C. albicans*, and another cytokine from the Th17 family, IL-23, increases angiogenesis and tumor growth, and antagonizes IL-12 and IFN.^47,48^ Another study showed that 54.2% of patients with gastric ulcer and 10.3% of patients with chronic gastritis had candidiasis, which was evident in 20% of patients with gastric cancer.^49,50^

## The role of fungal infections in colorectal cancer

CRC, the most common type of cancer worldwide, is the third leading cause of cancer and the fourth leading cause of cancer deaths, with a mortality rate of about 700 000. Overall, CRC accounts for 11% of all diagnosed cancers.^51^ By 2018, about 1.8 million new cases of CRC have been reported.^2^ The incidence of CRC in men is about 19.7 and in women 23.6 per 100 000 people and it is more common in developed countries.^52^ The mortality rate until 2018 was about 881 000.^2^ In general, mutations in oncogenes, tumor suppressor genes, and genes involved in DNA repair are associated with the development of CRC, and according to the primary origin of the mutation, CRCs are classified into three categories: *sporadic* (Most point mutations cause this type of CRC and occur in the genes APC [adenomatous polyposis coli], kRAS, Tp53, and DCC),^53^ *hereditary* (Most point mutations occur in another allele of the mutated gene, and this inherited form is divided into two groups: FAP (familial adenomatous polyposis) and HNPCC (Hereditary non-polyposis CRC due to mutations in DNA repair proteins such as MSH2, MLH1, MLH6, PMS1, and PMS2 occur),^54,55^ and *familial*. Environmental and hereditary risk factors play an important role in the development of CRC. CRC risk factors are classified into two categories: non-modifiable risk factors such as race,^56^ sex and age,^2^ IBD,^57^ cystic fibrosis,^58^ and cholecystectomy^59^ as well as modifiable risk factors such as obesity,^60^ diet,^61^ smoking and alcohol,^62^ and diabetes and resistance to insulin.^63^

The role of fungal agents in CRC has not been well understood due to low frequency and lack of reference genome, but using high-throughput sequencing methods, they have been able to identify several fungal species,^64^ and recently their role in IBD has been proven.^65^ Among the fungi that are associated with CRC, the following can be mentioned: Candida genus such as *C. tropicalis* and *C. albicans*, *Phoma,*^66^ *Malassezia,*^67^ *Trichosporon,*^68^ *Cladosporium*^69^ and so on. In addition to the above, fungi such as *Paracoccidioides*, *Histoplasma*, *Cryptococcus*, *Aspergillus*, *Penicillin*, *Zygomycetes*, *Pneumocystis*, *Scedosporiosis*cause colonic involvement and may eventually be involved in CRC formation.^70^ In this regard, a number of studies have revealed the relationship between fungal agents and CRC. According to a study by Luan et al, they examined fungal microbiota of biopsy specimens from intestinal adenomas and adjacent tissues using the deep sequencing technique and found that 45% of fungal microbiota contained the opportunistic pathogen *Candida* and *Phoma* and approximately 1% *Cladosporium*, *Trichosporon*, *Rhodotorula*, *Thanatephorus*, and *Plectosphaerella.*^66^ In another study by Gao in 2017, it was found that the ratio of ascomycota to basidiomycota was different in the three groups of control, polyp, and CRC, so that ascomycota has a frequency of 37-54% and basidiomycota 4-5%.^71^ In 2019, Coker et alalso examined colonic fungal microbiota, which are used as diagnostic markers for CRC, such as *A. flavus*, *Kwoniella mangrovensis*, *Pseudogymnoascus* sp. VKMF-4518, *Debaryomyces fabryi*, *A. sydowii*, *Moniliophthora perniciosa*,* K. heavenensis*, *A. ochraceoroseus*, *Talaromyces islandicus*,* Malassezia globosa*, *Pseudogymnoascus* sp. VKM F-4520, *A. rambellii*, *Pneumocystis murina*and *Nosema apis* and also found fungi such as *Malassezia*, *Moniliophthora*, *Rhodotorula*, *Acremonium*, *Thielaviopsis* and *Pisolithus* is associated with CRC.^72^

## The role of fungal infections in lung cancer

Lung cancer (LC) is one of the most common causes of cancer death in the world. LC incidence is 1.35 million new cases (representing 12.4% of all new cancer cases) and mortality is 1.18 million deaths (accounting for 17.6% of the global total).^26^ The five-year survival rate in newly diagnosed LC is 15%.^73^ Men are more likely to develop LC than women, and it affects more African American men.^74^ One of the major causes of LC is disruption of the molecular pathways of the lung cell, which alters normal cell growth, differentiation, and apoptosis.^75^ In general, LC is pathologically present in two forms: NSCLC (non-small cell lung carcinoma) (85% of cases) and SCLC (small-cell lung carcinoma) (15% of cases).^76^ Many internal and external factors are considered as risk factors of LC, including smoking, arsenic in drinking water, radioactive radon gas, asbestos, tuberculosis, COPD, HIV infection,^77^ and mutations in genes EGFR,^78^ BRAF,^79^ KRAS,^80^ and so on. As mentioned, one of the risk factors for LC is people with immunocompromised systems who become susceptible to lung disease and eventually LC by exposure to opportunistic fungi such as *Aspergillus* sp., *Cryptococcus* sp., *Pneumocystis* sp. and endemic fungi.^81^ For example, in opportunistic IPA (invasive pulmonary aspergillosis), people with immunocompromised systems are susceptible to *Aspergillus* and *Cryptococcus* and pose a serious threat to people with cancer.^82^ Thus, the differential diagnosis of LC solid tumors has revealed types of fungi such as *Trichosporon*, *Fusarium*, *Rhizopus*, *Histoplasma capsulatum*, *H. immitis*, and *Cryptococcus neoformans*. It has been shown that in patients with LC who receive corticosteroids, they open the way for the entry of opportunistic pathogens such as *Aspergillus* and *P. jiroveci* and exacerbate the disease.^83^ Another study reported that the dimorphic and opportunistic fungal pathogen *T. marneffei*, the causative agent of talaromycosis, causes lung infections in people with immunocompromised systems. Following a study by Ching-López and Rodríguez Pavón, a 56-year-old HIV-free woman was reported to have talaromycosis and co-infected with stage IV NSCLC. The patient received liposomal amphotericin B but she died due to a decrease in blood oxygen levels as well as a decrease in the level of blood cells.^84^ Another study by Watanabe et al was conducted in 2019 on a 69-year-old man who underwent chemoradiotherapy due to LC but later developed SIPA (subacute invasive pulmonary aspergillosis), a disease caused by *A. fumigatus*, due to immunocompromised system. For the treatment of fungal infections, he received amphotericin B in combination with voriconazole.^85^

## The role of fungal infections in cervical cancer

Cervical cancer (CC) is the third most common cancer of the female reproductive system in the world.^86^ According to a 2012 report, the standard rate for cervical cancer is about 14 cases per 100 000 people.^9^ In general, by 2018, about 530 000 women were diagnosed with CC, of which 257 000 died.^87^ The incidence of CC is higher in African and South Asian countries, and its incidence and mortality vary in most parts of the world. As a result, developed countries account for 86% of all CC cases and 88% of all CC deaths.^24,87^ The incidence rate of CC in Iran is about 2.61 per 100 000 people.^88^ The five-year survival rate in developed countries is about 66%.^89^ There are a number of risk factors for CC, including: reproductive factors, obesity, sexual behavior, diet, multiparity, prolonged use of hormonal contraceptives, poor socioeconomic status, smoking, Multiple pregnancies, and finally the presence of bacterial, viral and fungal infectious agents.^90^ A 2018 study identified several fungi from the cervicovaginal region, including *Candida*, *Malassezia*, *Sporidiobolaeae*, *Saccharomyces*, *Nakaseomyces*, *Gjaerumia*, and *Pleosporales.*^91^ According to a study by Moradi et al in Iran, one of the important fungi that is the link between cervicovaginal infections and cervical cancer was *C. albicans*, which was reported in women with high socioeconomic status in Bandar Abbas.^92^ In 2015, Neves et al studied a 42-year-old woman who had received immunosuppressive therapy for cervical neoplasia and developed fungemia due to *Cryptococcus laurentii* with a weakened immune system and was eventually treated with fluconazole.^93^

## The role of fungal infections in skin cancer

Skin cancer (SC) is one of the most common types of cancer among white populations and is generally classified into two categories: melanoma and non-melanoma (NMSC).^94^ According to the latest statistics from 2020, the number of new cases of skin cancer is estimated at 108 420 people and the approximate number of deaths is about 11 480 people.^95^ Remember that the increase in melanoma incidence is not parallel to the increase in mortality rates. NMSC, which includes Bowens disease, basal cell carcinoma (BCC), and squamous cell carcinoma (SCC), is more prevalent among Caucasian populations.^96^ Increased incidence of SC is associated with several factors including UV exposure, light skin (albinism), old age, male gender, chemicals (such as arsenic), radiation exposure, XP, weakened immune system, smoking, and viral (HPV) and fungal infections.^97^ Fungal infections have been shown to occur more frequently in people with weakened immune systems. Initially, most fungal infections were due to the presence of *Candida* species, but recently it has been shown that the frequency of *Aspergillus* sp. is increasing.^98^ In general, two types of fungal infections are involved in skin cancer: infections caused by pathogenic fungi such as *C. neoformans*, *H. capsulatum*, *C. immitis*, *Trichosporon*spp. etc., as well as opportunistic fungi such as *Candida* sp. (e.g., *C. albicans*, *C. tropicalis*, *C. glabrata*, *C. parapsilosis*), *Aspergillus, Mucorales, Fusarium, Rhizopus*.^99^ As mentioned, opportunistic fungal infections cause disease in people with weakened immune systems. In this regard, Pulido et al. in 2018 studied solid organ transplant recipients (SOTR) in such a way that by immunosuppression, graft survival is increased and this factor causes skin cancer and a variety of infections, followed by Those opportunistic infections are caused by black filamentous fungi such as *A. alternata*, *A. infectoria*, *C. cladosporioides*, *M. arundinis*, and *E. oligosperma.*^100^ Another study was conducted in 2016 by Brothers and Daveluy on a 61-year-old man whose immune system was suppressed due to receiving chemotherapy drugs for multiple myeloma. Following the injury, his finger became sore with a rose thorn and despite the weak immune system, the wound developed and squamous cell carcinoma developed and *C. parapsilosis* was isolated from the patient.^101^

## The role of fungal infections in ovarian cancer

Ovarian cancer (OC) is the seventh most common type of cancer in women in the world and after breast cancer is the second most common type of cancer in women over 40 (in a developed country).^102,103^ The highest incidence rates are observed in Eastern and Southern Europe and the lowest rates are observed in China.^104^ The five-year survival rate in the early stages of OC is about 29%, but when the tumor progresses it reaches about 92%.^105^ According to the latest statistics by 2020, the number of new OC cases is estimated at 21,750 and the number of deaths estimated at 13 940.^95^ Pathologically, OC is divided into three categories: epithelial OC (90% of cases), stromal OC (5-6% of cases), and genital OC (2-3% of cases).^106^ OC-related risk factors include: Reproductive risk factors (such as continuous ovulation)^107^ and hormonal factors (such as gonadotropins),^108^ family history and genetic predisposition (such as mutations in genes BRCA1, BRCA2 and MMR),^109^ ethnicity and race (such as Jews, Dutch, and French Canadians), diet (fiber and vitamin low D intake),^110^ lactation,^111^ obesity,^112^ smoking^113^ and alcohol consumption.^114^ Although fungal infections are not a risk factor for OC, a research team in 2017 studied the association between OC and fungal infections. According to the study, the following fungi were found in cancer samples: *Pneumocystis*, *Acremonium*, *Cladophialophora*, *Malassezia*, Microsporidia *Pleistophora*, *Ajellomyces, Aspergillus, Candida, Cladosporium, Coccidioides, Cryptococcus, Cunninghamella, Issatchenkia, Nosema, Paracoccidioides, Penicillium, Pleistophora, Rhizomucor, Rhizopus, Rhodotorula, Trichophyton.*^115^

## Results and Discussion

From the literature, it appears that fungal infections may play a significant role in the risk for precancerous lesions. The most common fungal species involved in cancers development are *Aspergillus flavus*, *A. parasiticus*, *Fusarium proliferatum*,* F. verticillioides*, *Candida albicans*, *C. tropicalis*,and *C. glabarata*.^115^ Furthermore, the ability of fungal species in carcinogens production such as acetaldehyde, nitrosamine, mycotoxins, and the generation of proinflammatory cytokines, can be risk factors in the promotion of various cancers.^26^

Moreover, the potential role of fungal infections in oncogenic processes is the subject of debate in recent surveys of this topic. There are suggestions that fungal infection is a cause of cancers development possibly when the infection is chronic and connected to risk factors such as alcohol, tobacco, and etc.; however, each class of cancer is influenced by different risk factors, such as family history, type 2 diabetes, and obesity, which are the main risk factors of cancer.^50,52^

Along with the increased incidence of cancer associated with fungal infections more thorough surveys of the epidemiology of cancer patients with fungal infections can help to determine which patients are most likely to be infected. Therefore, superior monitoring and diagnostic efforts can expand the accuracy of diagnosis of fungal infection and will increase patient outcomes by permitting intervention at an earlier stage of invasive cancers.^2,3^ The value of controlled clinical studies of patients with fungal infections will greatly increase our knowledge of the most operative methods of treating disseminated cancer disease in immune-compromised cancer patients.

## Conclusion

Several speciﬁc fungi are associated with carcinogenesis (Table 1). Recent studies indicated that the pathogenic fungal populations were increased in cancerous patients. It has been shown that the development of carcinogenesis is closely related to a fungal proﬁle. The most common genera of fungi that contribute to the development of versatile cancers are *Candida* sp., *Aspergillus* sp., and *Fusarium* sp. causing an inﬂammation and consequently contributing to the progression of various cancers. Although, the link between infectious fungi and carcinogenesis is not undeniable, we cannot restrict to the study of fungal microbiota. In fact, we need to explore the intimate connections that could exist with fungi and their possible role on carcinogenesis through their effect on the varied and complex immune system. It seems that the manipulation of fungal proﬁle can be a helpful approach in the rapid improvement of patients after anti-cancer treatment. Therefore, the focus on the potential effect of infectious fungi on the initiation of cancers and consequently the treatment of fungal infectious disease as a global approach is indispensable to predicate the possible subsequences of our current public health strategies.

**Table 1:** The latest clinical finding and the association of fungal infections with versatile cancers.

**Cancer type**	**Fungal agents**		**References**
Esophageal cancer	*Candida* sp.	*C. albicans, C. glabrata, C. tropicalis*	^16^
		*C. krusei, C. parapsilosis*	^116^
	*Torulopsis*sp.	*T. glabrata*, *T. tomata*	^17,117,118^
	*Aspergillus* sp.	*A. flavus*, *A. parasiticus*	^18-20,119^
	*Fusarium* sp.	*F. verticillioides*, *F. proliferation*	
Gastric cancer	*C. albicans, Phialemonium* spp., *Aspergillus* spp., *Penicillium* spp., *Fusarium* spp.		^37,38,39^
	*S. cerevisiae*, *Malassezia* spp., *Mucor* spp., *Cryptococcus* spp., *Rhodotorula* spp., *Trichosporon* spp., *Histoplasma* spp., *Coccidioides* spp., *Paracoccidioides* spp. and *Blastomyces* spp.		^40,41^
Gallbladder cancer	*Aspergillus* sp.	*A. flavus, A. parasiticus*	^39^ ^120,121^ ^122^ ^51^
	*Penicillium*		
	*Candida* sp.	*C. albicans*, *C. glabrata*, *C. tropicalis*	
	*Blastomyces dermatitidis, Cryptococcus neoformans*		
Colorectal cancer	*C. tropicalis*, *C. albicans*, *Phoma, Malassezia, Trichosporon, Cladosporium*		^66-69^ ^70^ ^66^ ^72^
	*Paracoccidioides*, *Histoplasma*, *Cryptococcus*, *Aspergillus*, *Penicillin*, *Zygomycetes*, *Pneumocystis*, *Scedosporiosis*		
	*Rhodotorula*, *Thanatephorus*, *Plectosphaerella*		
	*A. flavus*, *Kwoniella mangrovensis*, *Pseudogymnoascus* sp., *Debaryomyces fabryi*, *A. sydowii*, *Moniliophthora perniciosa*,* K. heavenensis*, *A. ochraceoroseus*, *Talaromyces islandicus*,* Malassezia globosa*,		
Colorectal cancer	*Pseudogymnoascus* sp., *A. rambellii*, *Pneumocystis murina, Nosema apis*, *Malassezia*, *Moniliophthora*, *Rhodotorula*, *Acremonium*, *Thielaviopsis*, *Pisolithus*		^72^
Lung cancer	*Aspergillus sp., Cryptococcus sp., Pneumocystis sp.*		^81^
	*Trichosporon*, *Fusarium*, *Rhizopus*, *Histoplasma capsulatum*, *H. immitis, P. jiroveci*		^83^
	*T. marneffei*		^84^
	*A. fumigatus*		^85^
Prostate cancer	*Candida sp.*, *Aspergillus sp.*, *C. neoformans*, *C. immitis*, *H. capsulatum*, *B. dermatitidis*		^123^
Cervical cancer	*Candida*, *Malassezia*, *Sporidiobolaeae*, *Saccharomyces*, *Nakaseomyces*, *Gjaerumia*, *Pleosporales*		^91^
	*Cryptococcus laurentii*		^93^
Skin cancer	*C. neoformans*, *H. capsulatum*, *C. immitis*, *Trichosporon*spp., *Mucor, Fusarium, Rhizopus*		^99^
	*Candida* sp., *C. albicans, C. tropicalis, C. glabrata, C. parapsilosis*		^99^
	*A. alternata*, *A. infectoria*, *C. cladosporioides*, *M. arundinis*, *E. oligosperma*		^100^
Breast cancer	*Aspergillus, Candida, Coccidioides, Cunninghamella, Geotrichum, Pleistophora, Rhodotorula, Filobasidiella, Mucor, Trichophyton, Epidermophyton, Fonsecaea, Pseudallescheria, Penicillium, Ajellomyces, Alternaria, Rhizomucor, Piedraia, Malassezia*		^124^
Ovarian cancer	Pneumocystis, Acremonium, Cladophialophora, Malassezia, Microsporidia Pleistophora, *Ajellomyces, Aspergillus, Candida, Cladosporium, Coccidioides, Cryptococcus, Cunninghamella, Issatchenkia, Nosema, Paracoccidioides, Penicillium, Pleistophora, Rhizomucor, Rhizopus, Rhodotorula, Trichophyton*		^115^

## Acknowledgments

 The authors acknowledge Faculty of Pharmacy, Tabriz University of Medical Sciences, Tabriz, Iran and Université de Paris, Faculté des Sciences, Paris, France.

 This work was supported and funded scheme by Tabriz University of Medical Sciences (PharmD. Thesis).

## Funding

 This work was supported by Tabriz University of Medical Sciences (Number: 52/6456), Tabriz, Iran.

## Ethical Issues

 Not applicable.

## Conflict of Interest

 All authors indicated that they have no conflicts of interest to disclose and are aware of its submission.‎
